# Exploring causal relationships between immune cells and age-related macular degeneration through univariable, bidirectional, and multivariable Mendelian analysis

**DOI:** 10.3389/fmed.2024.1444277

**Published:** 2024-12-24

**Authors:** Xixiang Wei, Hui Yang, Xue Yin, Zheng Fu, Weiwei Xiong

**Affiliations:** Department of Ophthalmology, Children's Hospital of Fudan University Xiamen Branch, Xiamen Children's Hospital, Xiamen, China

**Keywords:** causal relationship, Mendelian randomization, immune cells, age-related macular degeneration, GWAS

## Abstract

**Objective:**

This study systematically investigates the causal relationships between 731 immune cell phenotypes and age-related macular degeneration (AMD) using comprehensive Mendelian randomization (MR) analyses. The goal is to identify immune cell factors that contribute to or protect against AMD, thereby clarifying the immunological mechanisms underlying AMD pathophysiology and informing prevention and treatment strategies.

**Methods:**

Univariable, bidirectional, and multivariable MR analyses were conducted to evaluate the associations between immune cells and AMD. By utilizing publicly available GWAS datasets, we eliminated the need for individual consents. The large-scale MR approach adhered to STROBE-MR guidelines. Immune cell GWAS data were sourced from a study involving 3,757 Sardinians, encompassing a broad spectrum of immune phenotypes, while AMD summary statistics were derived from a GWAS with over 3,763 cases. Instrumental variables (IVs) were carefully selected to comply with MR assumptions, and multiple MR methods were employed to enhance the robustness of causal inferences. Additionally, we supplemented the data for dry AMD (2,469 cases and 206,221 controls) and wet AMD (2,114 cases and 206,601 controls) for validation purposes.

**Results:**

Univariable MR analysis identified 17 immune cell phenotypes significantly associated with AMD, including 11 potential risk factors and 6 potential protective factors. Bidirectional MR analysis found no significant effects of AMD on the examined immune cell subsets. Multivariable MR analysis indicated that TD CD4+ %T cells and CD39+ CD8br %T cells likely inhibit AMD development, whereas CD39+ CD8br %CD8br cells and CD45RA on resting Treg cells appear to increase AMD risk. Validation of immune cell subsets in dry and wet AMD revealed significant associations between specific immune cells and both forms of AMD, with some subsets uniquely linked to wet AMD and others to dry AMD.

**Conclusion:**

This study addresses a critical gap in understanding the causal relationship between immune cells and AMD, identifying immune cell subsets that may either mitigate or exacerbate AMD risk. Notably, it highlights the potential role of CD39+ CD8+ T cells as anti-inflammatory agents and potential targets for immunotherapy in AMD. The absence of bidirectional causality suggests a complex origin of immune dysregulation in AMD. The differential associations of immune cell subsets with AMD subtypes carry significant implications for precision medicine approaches in ophthalmology, laying a solid foundation for future research focused on understanding the immunological underpinnings of AMD and developing targeted therapeutic strategies.

## Introduction

Age-related macular degeneration (AMD) stands as the primary cause of vision impairment in Western societies (1), affecting approximately 21 million elderly individuals worldwide, a figure expected to surge due to population aging (2). AMD inflicts severe central vision loss, impacting about 11 million individuals in the United States and a staggering global prevalence of 170 million, projected to escalate to 288 million by 2040 (3, 4). The etiology of AMD, intricate in nature, involves environmental and genetic factors influencing susceptibility (1). While aging remains the chief risk factor for AMD, other contributors encompass family history, race, smoking, hypertension, and cataracts (5–7). Over the past two decades, mounting evidence has indicated a pivotal role for immune mechanisms in the pathobiology of AMD. Genome-wide association studies (GWAS) focusing on AMD have pinpointed specific genetic variants linked to diverse inflammatory pathways, notably encompassing complement genes and those governing the regulation of innate immune cells (8). Likewise, various omics disciplines, such as epigenomics, proteomics, and metabolomics, have elucidated immune-mediated pathways in the genesis of AMD (9, 10). These observations underscore the substantial impact of immune-mediated inflammation on the progression of AMD. Moreover, investigations have highlighted alterations in the expression of particular chemokine receptors on CD4+ T cells in AMD patients compared to healthy counterparts, suggesting a potential role for immune cells in the initiation and advancement of AMD through the secretion of chemokine receptors implicated in autoimmune and inflammatory disorders (11, 12).

Despite clinical observations and epidemiological data suggesting an association between immune cells and AMD, the causality of these associations and underlying biological mechanisms remain contentious. Furthermore, limitations of traditional observational studies, such as confounding variables and reverse causation, hinder accurate inference of causality. To address these limitations, Mendelian randomization (MR) offers a robust tool utilizing genetic variation as instrumental variables (IVs) to mitigate biases and confounding effects, thereby enhancing the accuracy of causal inference (13, 14).

Despite the widespread use of MR methods in causal inference in other disease domains, large-scale MR studies investigating the causal relationship between immune cells and AMD are lacking. This study aims to fill this gap by systematically investigating the associations between 731 immune cell phenotypes and AMD, employing univariable, bidirectional, and multivariable MR analyses. We anticipate that this study will provide new insights into the role of immune cells in AMD pathogenesis and inform scientific evidence for AMD prevention and treatment.

## Materials and methods

### Study design

**Figure 1:**
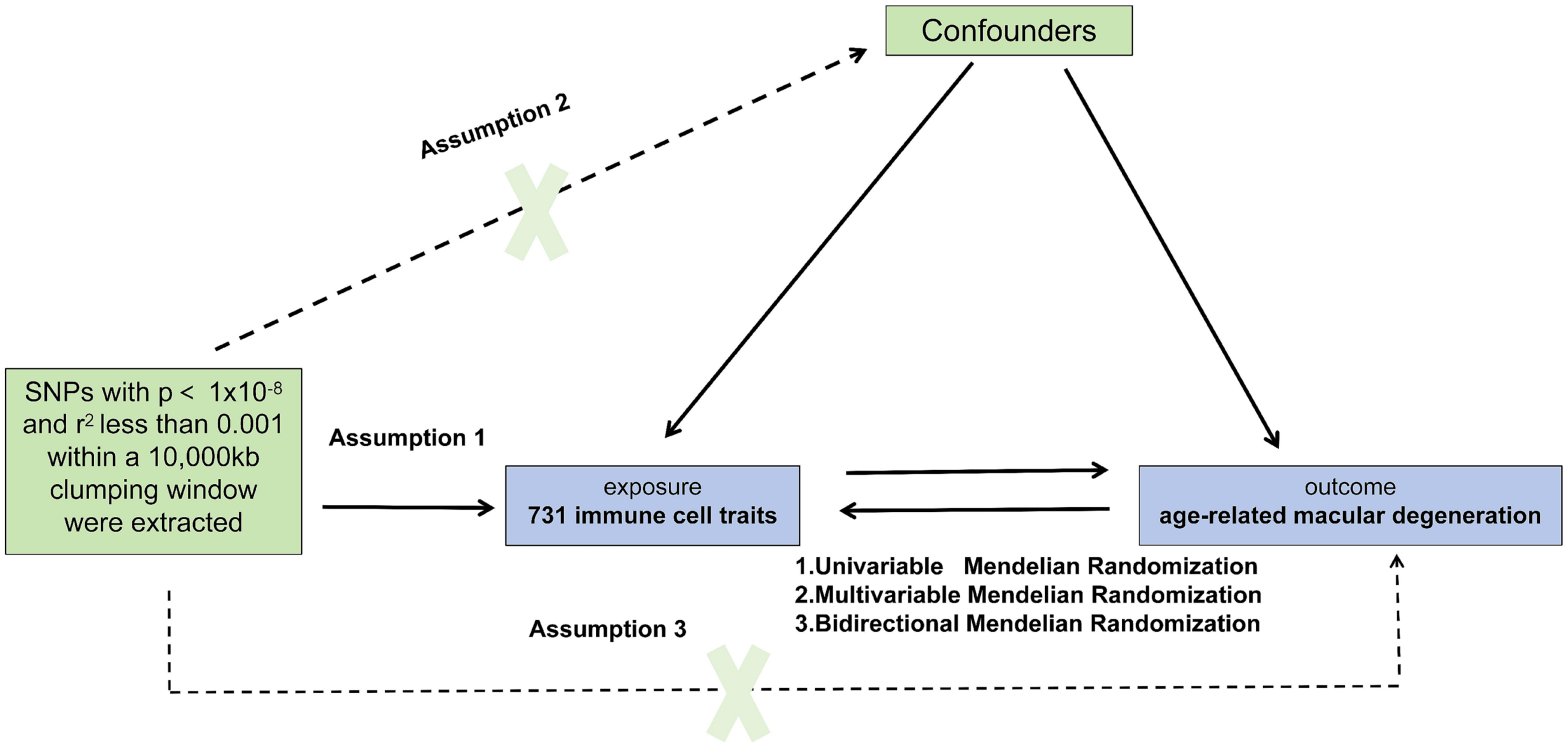
Causal relationship model between immune cells and Age-Related Macular Degeneration (AMD). This figure illustrates the framework for investigating the causal relationship between 731 immune cell types and AMD using Mendelian randomization. SNPs were extracted with *p* < 1 × 10^−8^ and r^2^ < 0.001 within a 10,000 kb clumping window. The model includes assumptions that SNPs directly influence immune cells (assumption 1), confounders may affect both immune cells and AMD (assumption 2), and SNPs indirectly influence AMD through immune cells (assumption 3). Three Mendelian randomization methods are applied: Univariable, Bidirectional, and Multivariable Mendelian Analysis.

Given the utilization of publicly accessible databases in our investigation, individual consent was not required. To explore the association between immune cells and AMD in the European population, we conducted univariable, bidirectional, and multivariable MR analyses. During MR analysis, IVs must meet three critical assumptions for reliable results: (1) a close association with the exposure, (2) independence from confounding factors influencing both the exposure and outcome, and (3) affecting the outcome solely through the exposure (15). To validate these assumptions, our study encompassed key steps: selection of genetic IVs linked to the exposure, utilization of various MR methods, and assessment of pleiotropy, heterogeneity, and sensitivity. We adhered to the latest STROBE-MR (16) guidelines. Figure 1 illustrates the MR study design.

### Data sources

#### GWAS data for immune cell

Data on immune cells were sourced from recent research involving 3,757 individuals of Sardinian ancestry from European populations. This study included 3,757 cases and 3,027 controls, with a gender distribution of 43% males and 57% females, aged 18 to 102 years. It assessed 731 immunophenotypes, covering 118 absolute cell counts, 389 median fluorescence intensities reflecting surface antigen levels, 32 morphological parameters, and 192 relative cell counts (Table 1). The study evaluated 22 million genetic variations (17).

**Table 1:** The GWAS data source details in our study.

Phenotype	Data source	Article	PMID	Case	Control	Sample size	Ancestry
731 immune cell traits	Orrù V et al.	Nat Genet	32929287	3,757	3,027	6,784	European
AMD (include dry and wet)	Finngen Biobank	–	–	3,763	205,359	209,122	European
Dry AMD	Finngen Biobank	–	–	2,469	206,221	208,690	European
Wet AMD	Finngen Biobank	–	–	2,114	206,601	208,715	European

#### GWAS data for AMD

The summary-level data for AMD were extracted from a large-scale mate-analysis GWAS including 3,763 cases and 205,359 controls and 16,380,424 variables from the FinnGen biobank analysis round 5. Additionally, we have also supplemented the data for dry AMD and wet AMD for validation purposes. The dry AMD data includes 2,469 cases and 206,221 controls; the wet AMD data includes 2,114 cases and 206,601 controls (Table 1).

#### Instrumental variable selection

Using R software, we selected single nucleotide polymorphisms (SNPs) associated with immune cells and AMD from GWAS data. Initially, we identified IVs significantly correlated with the exposure (*p* < 5 × 10^–8^), considering a distance threshold of 10,000 kb and an LD r2 threshold of 0.001 to mitigate linkage disequilibrium. SNPs incompatible with both exposure and outcome variables were eliminated. Subsequently, we normalized the SNPs concerning both the exposure and outcome variables, eliminating any incompatible or duplicated SNPs. Given that some IVs associated with the exposures might also be linked to age, smoking, diabetes, hypertension, and obesity, and could potentially confound the results, we queried all exposure IVs using PhenoScanner. However, we did not find any IVs strongly associated with age, smoking, diabetes, or hypertension among our selected IVs. Finally, to ensure consistency and remove any discrepancies, we harmonized palindromic and ambiguous SNPs with non-matching alleles between the exposure and outcome datasets. Palindromic SNPs are those for which the alleles are reverse complements of each other (e.g., A/G vs. A/C). Ambiguous SNPs are those with alleles that cannot be distinguished on the forward strand, such as A/T or C/G pairs (18). Harmonization was carried out by aligning the effect alleles and ensuring that the direction of the effect sizes matched between the exposure and outcome data. Additionally, to mitigate the influence of weak instrumental variables, we calculated the F-statistic using the formula F = β2exposure / SE2 exposure (19). If the F-statistic for the instrumental variables substantially surpasses 10, the risk of bias stemming from weak instrumental variables is deemed minimal (19).

#### MR method selection

We employed MR-Egger (20), weighted median (WM) (21), and inverse variance weighted (IVW) (22) techniques to determine the causal relationship between immune cells and AMD, with IVW as the primary analysis method. Cochran’s Q test assessed heterogeneity among SNPs, employing a fixed effects model unless heterogeneity (*p* < 0.05) warranted a random effects model. Sensitivity analysis involved weighted median and MR-Egger regression methods to test reliability and detect pleiotropy.

#### Sensitivity analysis

MR PRESSO detected SNP outliers, with subsequent exclusion if *p* < 0.01. The MR Egger intercept test examined horizontal pleiotropy, with an intercept of 0 and *p* > 0.05 indicating no horizontal pleiotropy. The “leave-one-out” method tested for SNP anomalies, considering *p* < 0.05 as indicative of potential causal relationships. All analyses were conducted using the “TwoSampleMR” package in R software version 4.1.2.

## Results

### Univariable MR

**Figure 2:**
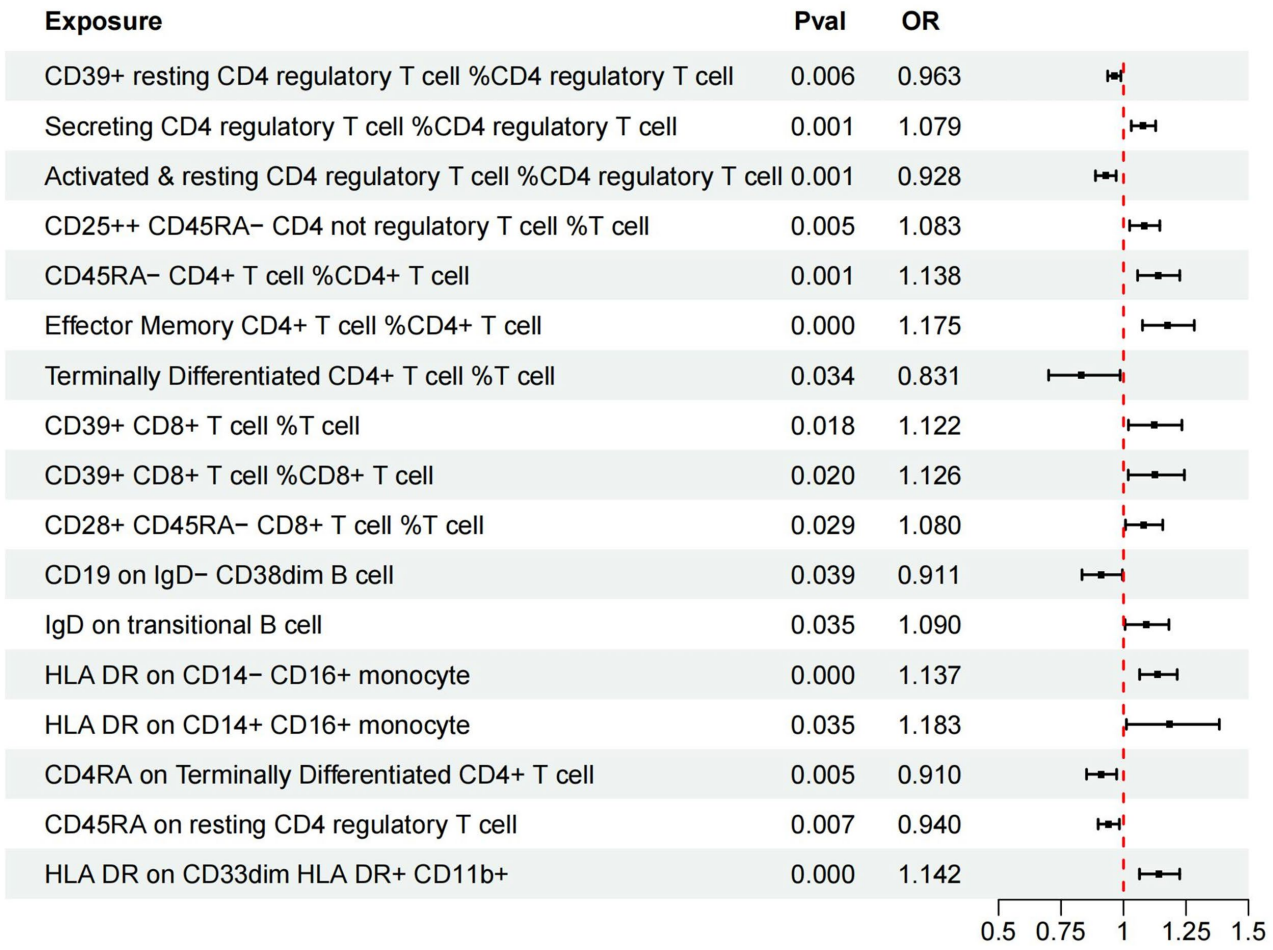
Forest plots of effects of immune cells on the AMD using univariable MR. AMD, Age-Related Macular Degeneration; IVW, inverse-variance weighted; OR, odds ratio; MR, Mendelian randomization.

In our investigation, we employed a two-sample MR analysis to explore the association between 731 immune cells and AMD. Employing the Inverse Variance Weighted (IVW) method, we identified 17 exposure factors significantly linked to AMD (PIVW <0.05) among the absolute counts of 731 immune cells, encompassing 11 risk factors and 6 protective factors (refer to Table 2 and Figure 2). Consequently, we identified 11 potential pathogenic factors and 6 potential protective factors among the immune cells associated with AMD. Notably, 11 immune cell subsets emerged as likely risk factors for AMD: Secreting Treg % CD4 Treg (OR = 1.079, 95% CI 1.031–1.129, *p* < 0.001), CD28+ CD45RA− CD8br %T cell (OR = 1.080, 95% CI 1.008–1.157, *p* = 0.029), CD25hi CD45RA− CD4 not Treg %T cell (OR = 1.083, 95% CI 1.025–1.145, *p* = 0.005), IgD on transitional (OR = 1.090, 95% CI 1.006–1.182, *p* = 0.035), CD39+ CD8br %T cell (OR = 1.122, 95% CI 1.020–1.234, *p* = 0.018), CD39+ CD8br %CD8br (OR = 1.126, 95% CI 1.019–1.244, *p* = 0.02), HLA DR on CD14− CD16+ monocyte (OR = 1.137, 95% CI 1.064–1.215, *p* < 0.001), CD45RA− CD4+ %CD4+ (OR = 1.138, 95% CI 1.056–1.225, *p* = 0.001), HLA DR on CD33dim HLA DR+ CD11b+ (OR = 1.142, 95% CI 1.064–1.225, *p* < 0.001), EM CD4+ %CD4+ (OR = 1.175, 95% CI 1.076–1.283, *p* < 0.001), HLA DR on CD14+ CD16+ monocyte (OR = 1.183, 95% CI 1.012–1.383, *p* = 0.035). Conversely, 6 immune cell features may mitigate the risk of AMD: TD CD4+ %T cell (OR = 0.831, 95% CI 0.700–0.987, *p* = 0.034), CD4RA on TD CD4+ (OR = 0.910, 95% CI 0.852–0.972, *p* = 0.005), CD19 on IgD− CD38dim (OR = 0.911, 95% CI 0.834–0.995, *p* = 0.039), Activated & resting Treg % CD4 Treg (OR = 0.928, 95% CI 0.888–0.970, *p* < 0.001), CD45RA on resting Treg (OR = 0.940, 95% CI 0.899–0.984, *p* = 0.007), CD39+ resting Treg % CD4 Treg (OR = 0.963, 95% CI 0.937–0.989, *p* = 0.006).

**Table 2:** Univariable MR estimates from IVW methods of assessing the causal effect of immune cell on AMD.

Exposure	Panel	MR methods	OR (95% CI)	*p*-value
CD39+ resting Treg % CD4 Treg	Treg	IVW	0.963 (0.937–0.989)	0.006
Secreting Treg % CD4 Treg	Treg	IVW	1.079 (1.031–1.129)	0.001
Activated and resting Treg % CD4 Treg	Treg	IVW	0.928 (0.888–0.970)	0.001
CD25hi CD45RA− CD4 not Treg %T cell	Treg	IVW	1.083 (1.025–1.145)	0.005
CD45RA− CD4+ %CD4+	Maturation stages of T cell	IVW	1.138 (1.056–1.225)	0.001
EM CD4+ %CD4+	Maturation stages of T cell	IVW	1.175 (1.076–1.283)	<0.001
TD CD4+ %T cell	Maturation stages of T cell	IVW	0.831 (0.700–0.987)	0.034
CD39+ CD8br %T cell	Treg	IVW	1.122 (1.020–1.234)	0.018
CD39+ CD8br %CD8br	Treg	IVW	1.126 (1.019–1.244)	0.02
CD28+ CD45RA− CD8br %T cell	Treg	IVW	1.080 (1.008–1.157)	0.029
CD19 on IgD− CD38dim	B cell	IVW	0.911 (0.834–0.995)	0.039
IgD on transitional	B cell	IVW	1.090 (1.006–1.182)	0.035
HLA DR on CD14− CD16+ monocyte	Monocyte	IVW	1.137 (1.064–1.215)	<0.001
HLA DR on CD14+ CD16+ monocyte	Monocyte	IVW	1.183 (1.012–1.383)	0.035
CD4RA on TD CD4+	Maturation stages of T cell	IVW	0.910 (0.852–0.972)	0.005
CD45RA on resting Treg	Treg	IVW	0.940 (0.899–0.984)	0.007
HLA DR on CD33dim HLA DR+ CD11b+	Myeloid cell	IVW	1.142 (1.064–1.225)	<0.001

IVW, inverse-variance weighted; OR, odds ratio; CI, confidence interval; MR, Mendelian randomization; AMD, Age-Related Macular Degeneration.

### Bidirectional MR

Our results did not demonstrate any significant impact of AMD on the studied immune cell subsets. The *p*-values obtained from the instrumental variable weighted (IVW) method ranged from 0.18 to 0.98, indicating no statistically significant causal relationship between AMD and the immune cell subsets analyzed (Table 3).

**Table 3:** Bidirectional MR estimates from IVW methods of assessing the causal effect of immune cell on AMD.

Exposure	Outcome	MR methods	OR (95% CI)	*p*-value
AMD	CD39+ resting Treg % CD4 Treg	IVW	1.012 (0.957 to 1.070)	0.67
AMD	Secreting Treg % CD4 Treg	IVW	0.989 (0.944 to 1.035)	0.63
AMD	Activated & resting Treg % CD4 Treg	IVW	1.012 (0.965 to 1.060)	0.63
AMD	CD25hi CD45RA− CD4 not Treg %T cell	IVW	0.967 (0.913 to 1.025)	0.26
AMD	CD45RA− CD4+ %CD4+	IVW	1.002 (0.959 to 1.047)	0.92
AMD	EM CD4+ %CD4+	IVW	1.019 (0.974 to 1.065)	0.42
AMD	TD CD4+ %T cell	IVW	1.019 (0.966 to 1.076)	0.49
AMD	CD39+ CD8br %T cell	IVW	0.999 (0.953 to 1.048)	0.98
AMD	CD39+ CD8br %CD8br	IVW	0.980 (0.933 to 1.028)	0.41
AMD	CD28+ CD45RA− CD8br %T cell	IVW	1.005 (0.956 to 1.056)	0.85
AMD	CD19 on IgD− CD38dim	IVW	0.996 (0.949 to 1.046)	0.89
AMD	IgD on transitional	IVW	1.004 (0.955 to 1.055)	0.88
AMD	HLA DR on CD14− CD16+ monocyte	IVW	1.013 (0.950 to 1.079)	0.70
AMD	HLA DR on CD14+ CD16+ monocyte	IVW	1.024 (0.976 to 1.074)	0.33
AMD	CD4RA on TD CD4+	IVW	0.972 (0.924 to 1.023)	0.27
AMD	CD45RA on resting Treg	IVW	0.968 (0.922 to 1.015)	0.18
AMD	HLA DR on CD33dim HLA DR+ CD11b+	IVW	1.043 (0.961 to 1.132)	0.31

IVW, inverse-variance weighted; OR, odds ratio; CI, confidence interval; MR, Mendelian randomization; AMD, Age-Related Macular Degeneration.

### Multivariable MR

**Figure 3:**
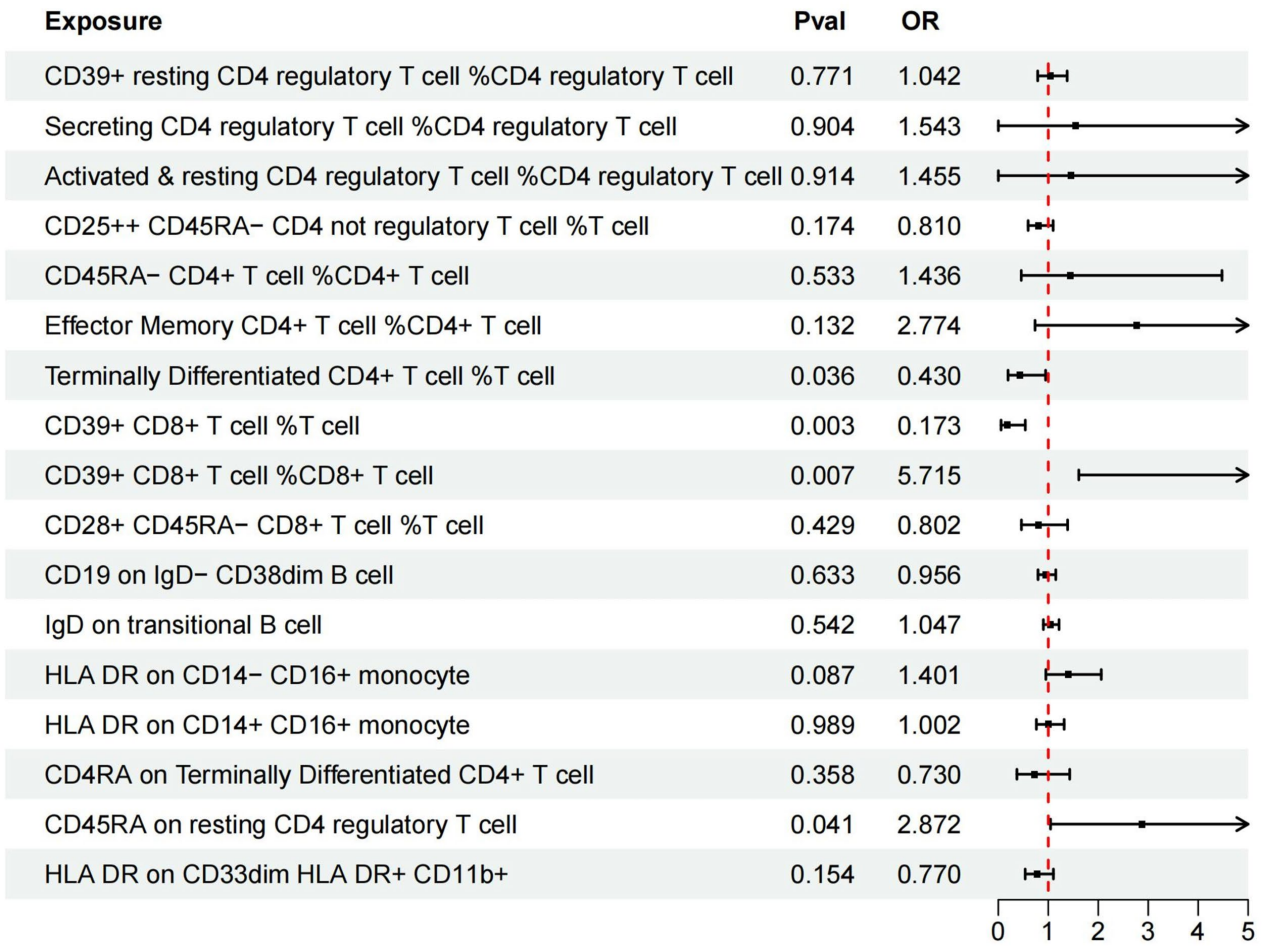
Forest plots of effects of immune cells on the AMD using multivariable MR. AMD, Age-Related Macular Degeneration; IVW, inverse-variance weighted; OR, odds ratio; MR, Mendelian randomization.

Additionally, we conducted multivariable MR analysis on the 17 exposure factors significantly associated with AMD, identifying four exposure factors significantly linked to AMD (*p* < 0.05). Among them, TD CD4+ %T cell (OR = 0.430, 95% CI 0.195–0.947, *p* = 0.036) and CD39+ CD8br %T cell (OR = 0.173, 95% CI 0.055–0.541, *p* = 0.003) may mitigate the risk of AMD, while CD39+ CD8br %CD8br (OR = 5.715, 95% CI 1.611–20.273, *p* = 0.007) and CD45RA on resting Treg (OR = 2.872, 95% CI 1.046–7.884, *p* = 0.041) are more likely to be risk factors for AMD (refer to Table 4 and Figure 3).

**Table 4:** Multivariable MR estimates from IVW methods of assessing the causal effect of immune cell on AMD.

Exposure	Panel	MR methods	OR (95% CI)	*p*-value
CD39+ resting Treg % CD4 Treg	Treg	IVW	1.042 (0.788–1.378)	0.771
Secreting Treg % CD4 Treg	Treg	IVW	1.543 (0.001–1752.152)	0.904
Activated & resting Treg % CD4 Treg	Treg	IVW	1.455 (0.002–1282.258)	0.914
CD25hi CD45RA− CD4 not Treg %T cell	Treg	IVW	0.810 (0.597–1.098)	0.174
CD45RA− CD4+ %CD4+	Maturation stages of T cell	IVW	1.436 (0.461–4.476)	0.533
EM CD4+ %CD4+	Maturation stages of T cell	IVW	2.774 (0.736–10.462)	0.132
TD CD4+ %T cell	Maturation stages of T cell	IVW	0.430 (0.195–0.947)	0.036
CD39+ CD8br %T cell	Treg	IVW	0.173 (0.055–0.541)	0.003
CD39+ CD8br %CD8br	Treg	IVW	5.715 (1.611–20.273)	0.007
CD28+ CD45RA− CD8br %T cell	Treg	IVW	0.802 (0.463–1.387)	0.429
CD19 on IgD− CD38dim	B cell	IVW	0.956 (0.795–1.150)	0.633
IgD on transitional	B cell	IVW	1.047 (0.902–1.216)	0.542
HLA DR on CD14− CD16+ monocyte	Monocyte	IVW	1.401 (0.952–2.061)	0.087
HLA DR on CD14+ CD16+ monocyte	Monocyte	IVW	1.002 (0.762–1.317)	0.989
CD4RA on TD CD4+	Maturation stages of T cell	IVW	0.730 (0.372–1.429)	0.358
CD45RA on resting Treg	Treg	IVW	2.872 (1.046–7.884)	0.041
HLA DR on CD33dim HLA DR+ CD11b+	Myeloid cell	IVW	0.770 (0.538–1.103)	0.154

IVW, inverse-variance weighted; OR, odds ratio; CI, confidence interval; MR, Mendelian randomization; AMD, Age-Related Macular Degeneration.

The shaded colored sections represent the rows with a *p*-value < 0.05.

### Sensitivity analysis

#### Univariable MR

For a comprehensive presentation of all results in this section, refer to Table 5. No heterogeneity was observed across the exposures to the 17 types of immune cells. MR-Egger regression indicated no horizontal pleiotropy for any exposure. No outliers were identified, and the direction of results from MR-Egger and weighted median (WM) analyses was consistent with IVW results (see Supplementary Figure S1). Leave-one-out analysis further confirmed the stability of the results (see Supplementary Figure S2). Consequently, we consider the results obtained via IVW to be reliable.

**Table 5:** Sensitivity analysis of immune cell causally linked to AMD.

Exposures	Outcomes	Heterogeneity test				Pleiotropy test		MR-PRESSO
		IVW		MR-Egger		MR-Egger intercept		
		Q	*p*-value	Q	*p*-value	Intercept	*P*	*p*
CD39+ resting Treg % CD4 Treg	AMD	14.72	0.40	13.39	0.42	0.01984	0.28	0.47
Secreting Treg % CD4 Treg	AMD	10.04	0.19	9.69	0.14	−0.01499	0.66	0.40
Activated & resting Treg % CD4 Treg	AMD	10.04	0.19	9.72	0.14	0.01426	0.67	0.43
CD25hi CD45RA− CD4 not Treg %T cell	AMD	6.88	0.08	4.63	0.10	−0.20513	0.43	0.37
CD45RA− CD4+ %CD4+	AMD	4.12	0.39	4.12	0.25	−0.00080	0.98	0.61
EM CD4+ %CD4+	AMD	1.49	0.69	1.46	0.48	−0.00607	0.87	0.83
TD CD4+ %T cell	AMD	15.75	0.01	14.83	0.01	−0.03109	0.65	0.08
CD39+ CD8br %T cell	AMD	3.35	0.76	3.01	0.70	−0.02405	0.59	0.83
CD39+ CD8br %CD8br	AMD	3.31	0.65	2.93	0.57	−0.02684	0.57	0.73
CD28+ CD45RA− CD8br %T cell	AMD	4.57	0.21	2.05	0.36	0.16808	0.26	0.48
CD19 on IgD− CD38dim	AMD	2.37	0.50	1.29	0.52	0.03165	0.41	0.49
IgD on transitional	AMD	3.87	0.42	2.27	0.52	−0.04233	0.30	0.46
HLA DR on CD14− CD16+ monocyte	AMD	8.29	0.31	7.93	0.24	−0.01934	0.62	0.29
HLA DR on CD14+ CD16+ monocyte	AMD	21.92	0.00	20.79	0.00	−0.06061	0.67	0.01
CD4RA on TD CD4+	AMD	8.59	0.07	7.11	0.07	0.03847	0.49	0.27
CD45RA on resting Treg	AMD	11.80	0.11	11.64	0.07	−0.00974	0.78	0.19
HLA DR on CD33dim HLA DR+ CD11b+	AMD	7.61	0.11	7.35	0.06	−0.03368	0.77	0.17

### Validation of immune cell subsets in dry and wet AMD

Table 6 presents the results of the univariable MR analysis conducted using the inverse variance weighted (IVW) method. This analysis assesses the potential causal effects of various immune cell subsets on both dry (DRY) and wet (WET) forms of AMD.

**Table 6:** Univariable MR estimates from IVW methods of assessing the causal effect of immune cell on DRY AMD and WET AMD.

Exposure	Panel	Method	DRY AMD		WET AMD	
			OR (95% CI)	*p*-value	OR (95% CI)	*p*-value
CD39+ resting Treg % CD4 Treg	Treg	IVW	0.958 (0.947, 0.968)	0.01	0.962 (0.927, 0.997)	0.032
Secreting Treg % CD4 Treg	Treg	IVW	1.081 (1.037, 1.127)	0.005	1.101 (1.044, 1.161)	0.001
Activated & resting Treg % CD4 Treg	Treg	IVW	0.927 (0.883, 0.972)	0.005	0.909 (0.855, 0.965)	0.001
CD45RA− CD4+ %CD4+	Maturation stages of T cell	IVW	1.133 (1.051, 1.222)	0.006	1.183 (1.085, 1.292)	0.001
EM CD4+ %CD4+	Maturation stages of T cell	IVW	1.160 (1.068, 1.263)	0.006	1.242 (1.131, 1.368)	0.001
HLA DR on CD14− CD16+ monocyte	Monocyte	IVW	1.105 (1.038, 1.182)	0.014	1.208 (1.126, 1.297)	0.001
HLA DR on CD14+ CD16+ monocyte	Monocyte	IVW	1.185 (1.054, 1.333)	0.022	1.224 (1.107, 1.365)	0.007
CD25hi CD45RA− CD4 not Treg %T cell	Treg	IVW	1.096 (1.062–1.202)	0.022	1.086 (0.956–1.233)	0.207
TD CD4+ %T cell	Maturation stages of T cell	IVW	0.842 (0.678–1.046)	0.121	0.77 (0.665–0.893)	0.001
CD39+ CD8br %T cell	Treg	IVW	1.107 (0.987–1.242)	0.082	1.14 (1.007–1.29)	0.038
CD39+ CD8br %CD8br	Treg	IVW	1.112 (0.986–1.254)	0.083	1.135 (1.182–1.293)	0.045
CD28+ CD45RA− CD8br %T cell	Treg	IVW	1.054 (0.959–1.159)	0.276	1.108 (1.013–1.213)	0.025
CD19 on IgD− CD38dim	B cell	IVW	0.935 (0.837–1.044)	0.234	0.9 (0.802–0.97)	0.032
IgD on transitional	B cell	IVW	1.069 (0.959–1.192)	0.226	1.067 (1.041–1.185)	0.023
CD4RA on TD CD4+	Maturation stages of T cell	IVW	0.913 (0.841–0.992)	0.031	0.909 (0.753–1.097)	0.319
CD45RA on resting Treg	Treg	IVW	0.959 (0.892–1.031)	0.261	0.909 (0.85–0.972)	0.005
HLA DR on CD33dim HLA DR+ CD11b+	Myeloid cell	IVW	1.125 (0.972–1.301)	0.114	1.188 (1.046–1.35)	0.008

AMD, Age-Related Macular Degeneration; IVW, inverse-variance weighted; OR, odds ratio; CI, confidence interval; MR, Mendelian randomization.

The cells filled with pink represent *p*-values < 0.05, while the cells filled with light green represent *p*-values > 0.05.

The results reveal that a significant majority of immune cells previously identified with statistically significant associations are linked to both DRY and WET AMD. Notably, some immune cells are specifically associated with WET AMD, whereas only a small fraction is related to DRY AMD. These findings highlight the intricate interplay between immune cell subsets and the development of AMD, suggesting potential targets for therapeutic intervention.

## Discussion

In this study, we utilized univariable, bidirectional, and multivariable MR approaches to explore the relationship between 731 immune cells and AMD. By leveraging extensive publicly available GWAS summary data, we elucidated the intricate interplay between immune cells and AMD, with sensitivity analyses confirming the robustness of our findings. Our univariable MR analysis identified 11 potential pathogenic factors and 6 potential protective factors associated with AMD. Moreover, bidirectional MR analysis revealed no evidence of bidirectional causality between immune cells and AMD. Finally, our multivariable MR analysis demonstrated that, after adjusting for the effects of other immune cells, TD CD4+ %T cells and CD39+ CD8br %T cells exerted inhibitory effects on AMD development, while CD39+ CD8br %CD8br and CD45RA expression on resting Tregs promoted AMD progression.

Additionally, our study enhances the understanding of the immunological landscape in AMD by validating the causal relationships between specific immune cell subsets and the development of both dry (DRY) and wet (WET) forms of AMD. The analysis highlighted the heterogeneity in immune responses, with certain subsets specifically linked to WET AMD and others to DRY AMD, emphasizing the necessity for tailored therapeutic strategies. Furthermore, the differential associations of immune cell subsets with AMD subtypes carry significant implications for precision medicine approaches in ophthalmology.

### Univariable MR

In our investigation, we employed MR methods to explore potential causal links between immune cells and AMD. We identified a range of immune cells and associated factors linked to the disease, some of which may exert pathogenic effects, while others could confer protective benefits. Our results unveiled significant associations between distinct immune cell types and AMD, underscoring the contributions of various immune cells, including T cells, B cells, and monocytes, to the initiation and progression of the condition.

Initially, we observed a negative correlation between the frequency of specific immune cell subsets and AMD occurrence. Notably, the expression levels of CD4RA in CD4+ T cells and certain B cell subtypes, such as IgD− CD38dim, exhibited negative associations with AMD development, hinting at potential protective roles for these immune cells in mitigating or preventing AMD progression.

Conversely, positive correlations emerged between certain immune cell subsets and AMD. For instance, certain T cell subsets, notably CD4+ T cells and CD8br T cells expressing CD39+, showed positive associations. Studies have reported the presence of CD8+ T cells in the choroid of AMD patients with GA (23) and NVAMD (24) using fluorescence microscopy on frozen sections, along with elevated levels of IL-17 and IL-22, T17 cytokines, in the plasma of AMD patients compared to non-AMD controls (25, 26). This elevation may be partly driven by the activation of the complement component C5a stimulating CD4+ T cells (26). Furthermore, specific monocyte subsets, such as CD14− CD16+ and CD14+ CD16+ monocytes expressing HLA DR, exhibited positive correlations with AMD. Monocytes represent circulating innate immune cells in the blood, originating from the bone marrow, while macrophages serve as tissue-resident phagocytic innate immune cells, playing pivotal roles (27, 28). In humans, these subsets encompass classical CD14++CD16−, intermediate CD14++CD16+, and nonclassical CD14 + CD16+ monocytes (29, 30). Nonetheless, our understanding of these subsets’ biology in humans remains limited, with research suggesting potential pro-inflammatory effects for nonclassical and intermediate subsets, contrasting with the more reparative functions of the classical subset (29, 30). These implications suggest possible involvement of these immune cells in AMD pathogenesis, potentially fostering the disease through immune-inflammatory pathways.

Moreover, we observed statistically significant correlations between specific cell subsets and AMD. For example, the expression levels of CD28+ CD45RA− CD8br in certain T cell subsets and the frequency of CD25hi CD45RA− CD4 non-regulatory T cells were significantly positively correlated with AMD. Another study reported an increase in the percentage of CD56+ and CD28− memory T cells in the blood associated with an increased risk of developing AMD (31), with further increased risk observed for patients carrying at least one CFH H402 allele (31). Additionally, a lower percentage of CD8+ CXCR3 T cells and CD4+ CD69+ CXCR3+ T cells was observed in NVAMD compared to controls (32). This underscores the immune system’s importance in AMD pathogenesis.

### Bidirectional MR

Our bidirectional MR analysis did not identify significant impacts of AMD on immune cells, suggesting that the development of AMD may not directly influence immune cell levels or functions. This challenges previous hypotheses of a bidirectional relationship, indicating that immune dysregulation in AMD may primarily arise from factors such as genetic predispositions or environmental influences, rather than the disease itself.

### Multivariable MR

This study employed MR to investigate the causal connections between 731 types of immune cells and AMD, aiming to discern potential pathogenic and protective factors. Preliminary analysis unveiled 11 potential pathogenic factors and 6 potential protective factors. Subsequently, we conducted multivariable MR analysis on these 17 candidate factors to ascertain their causal links with AMD. This method effectively minimized the influence of confounding variables, thereby bolstering the robustness of our findings. Our analysis revealed four immune cell characteristics significantly associated with AMD, offering novel insights into AMD’s immunopathological mechanisms.

A significant discovery was the negative association between the expression of CD39+ CD8br %T cells and AMD risk (OR = 0.173, 95% CI: 0.055–0.541, *p* = 0.003), implying a protective role for these cells in AMD development. CD39, an immunoregulatory enzyme, is known for its anti-inflammatory properties, and decreased expression on T cells may attenuate inflammatory damage in AMD. Conversely, the expression of CD39+ CD8br %CD8br was positively correlated with AMD risk (OR = 5.715, 95% CI: 1.611–20.273, *p* = 0.007), suggesting the intricate involvement of distinct CD8+ T cell subpopulations in AMD pathogenesis (12).

Furthermore, TD CD4+ %T cells exhibited a significant negative correlation with AMD (OR = 0.430, 95% CI: 0.195–0.947, *p* = 0.036), indicating a protective function. These cells may modulate immune responses, potentially reducing AMD risk. Notably, research suggests that CCR6, a chemokine receptor, could influence GA progression, making it a promising molecular target for further exploration. It is plausible that TD CD4+ %T cells regulate AMD progression via CCR6 secretion (11).

Finally, heightened expression of CD45RA on resting regulatory T cells (Tregs) was associated with increased AMD risk (OR = 2.872, 95% CI: 1.046–7.884, *p* = 0.041), suggesting that alterations in Treg status may impact AMD development. CD45RA-expressing resting Tregs might exert pro-inflammatory effects, thereby elevating AMD risk. Tregs typically maintain immune tolerance, and changes in CD45RA expression could affect their function, potentially influencing AMD pathogenesis. Studies indicate that Tregs in PCV patients, a condition linked to AMD, exhibit decreased percentages and a more Th2-like polarization compared to healthy controls (33), indicating their potential role in AMD’s immunopathogenesis.

### Validation of immune cell subsets in dry and wet AMD

The findings from our MR analysis reveal a complex interplay between immune cells and AMD, with certain subsets demonstrating unique associations with DRY and WET AMD. For instance, the negative association observed for CD39+ resting Treg % CD4 Treg with both forms of AMD suggests a potential protective role for this immune cell subset in AMD development. This aligns with the known immunomodulatory functions of Tregs, which are capable of suppressing excessive immune responses that may contribute to AMD progression.

Conversely, the positive association of Secreting Treg % CD4 Treg with both forms of AMD implies a potential pathogenic role, reflecting the delicate balance between pro-inflammatory and regulatory immune responses in the pathogenesis of AMD. The contrasting effects of different Treg subsets highlight the complexity of immune regulation in AMD and the potential for targeted therapeutic interventions.

The observation that certain immune cell subsets are specifically associated with WET AMD, while others relate to DRY AMD, underscores the heterogeneity in immune responses between the two subtypes. For example, the absence of a significant association for TD CD4+ % T cells with DRY AMD, coupled with a significant reduction in risk for WET AMD, suggests that this subset may play a more pronounced role in the neovascularization process characteristic of WET AMD. This finding is consistent with previous research indicating distinct immunological signatures in neovascular AMD, emphasizing the need for tailored therapeutic approaches for different AMD subtypes.

The differential associations of immune cell subsets with DRY and WET AMD have important implications for the development of precision medicine strategies in ophthalmology. By identifying immune cell subsets specifically associated with each AMD subtype, we can gain deeper insights into the underlying immunopathological mechanisms and potentially develop targeted therapies that modulate these immune cells to prevent or treat AMD more effectively.

While our study offers novel insights into the immune cell subsets associated with AMD, it is not without limitations. The generalizability of our findings may be constrained due to the reliance on data primarily from European populations. Additionally, while MR analysis aids in establishing causality, it does not provide direct evidence of the biological mechanisms involved. Furthermore, the study’s focus on specific immune cell phenotypes may overlook the broader immune response dynamics that could also play a role in AMD pathogenesis. Future research should aim to validate these findings in diverse populations and investigate the functional roles of these immune cells within the AMD microenvironment. Further mechanistic studies are also essential to unravel the complex interactions between immune cells and the pathogenesis of AMD, which will be crucial for developing effective immunotherapies.

## Conclusion

Our study emphasizes the significance of immune cell subsets in the pathogenesis of AMD and highlights the potential for precision medicine approaches in managing this condition. The validation of these immune cell subsets in DRY and WET AMD lays a solid foundation for future research focused on understanding the immunological underpinnings of AMD and developing targeted therapeutic strategies.

## Data Availability

The original contributions presented in the study are included in the article/Supplementary material, further inquiries can be directed to the corresponding author.
