# Supplementary material for: Exploring causal relationships between immune cells and age-related macular degeneration through univariable, bidirectional, and multivariable Mendelian analysis
Source: Front Med (Lausanne). 2024 Dec 24;11:1444277. doi: 10.3389/fmed.2024.1444277 (PMC11703709; doi:10.3389/fmed.2024.1444277)
Supplement: Supplementary file 1 [file Data_Sheet_1.docx]

Supplementary Figure1.Scatterplots for MR analyses of the causal effect of immune cells on the AMD. The slope of each line corresponds to the estimated MR effect per method.





Supplementary Figure2.Leave-one-out analysis of the causal effect of immune cells on the AMD. Each black point represents the IVW MR method applied to estimate the causal effect of immune cells on the AMD, excluding that particular variant from the analysis. The red point represents the IVW estimate using all SNPs.
